# Pharmacy employees’ involvement in safeguarding persons with dementia who use dietary supplements: Results from a survey of Norwegian pharmacies

**DOI:** 10.1186/s12906-019-2587-4

**Published:** 2019-07-19

**Authors:** Hilde Risvoll, Frauke Musial, Kjell H. Halvorsen, Trude Giverhaug, Marit Waaseth

**Affiliations:** 1 NAFKAM, Institute for Community Medicine, UiT The Arctic University of Norway, pb 6050 Langnes, 9037 Tromsø, Norway; 2 NKS Kløveråsen as, Junkernveien 67, 8076 Bodø, Norway; 3 Valnesfjord Helsesportssenter, Østerkløftveien 249, 8215 Valnesfjord, Norway; 4 Department of Pharmacy, UiT The Arctic University of Norway, pb 6050 Langnes, 9037 Tromsø, Norway; 5 RELIS North Norway, University Hospital of North Norway, Sykehusvegen 38, 9019 Tromsø, Norway

**Keywords:** Pharmacy, Dietary supplements, Dementia, Patient safety, Risk management, Professional practice behavior, Attitude, Attributed responsibility, Cross-sectional survey

## Abstract

**Background:**

Community-dwelling persons with dementia commonly use dietary supplements (DS), often without receiving help with the administration. Patient safety is a concern, as DS-drug interactions and adverse events are potential complications. Since many persons with dementia buy their DS in pharmacies, we investigated Norwegian pharmacy employees’ attitudes and professional practice behaviors related to DS.

**Methods:**

We conducted a survey in eight Norwegian municipalities of pharmacy employees involved in the sale of DS. The questionnaire covered demographics and investigated attitudes toward DS, professional practice behaviors related to the sale of DS, experiences with customers with dementia, and perceived and attributed responsibilities with regard to patient safety.

**Results:**

One hundred and five employees responded (response rate: 52%). Most employees regarded general practitioners (GPs) as primarily responsible for safeguarding the use of DS by persons with dementia and rated themselves less responsible. Thirty-seven percent of the employees reported personal use of DS (past or current use). Nine percent considered some of the DS to have symptomatic or prophylactic effects against dementia. Forty-eight percent confirmed that they informed customers about potential adverse events; 42% indicated that they did this sometimes. Sixteen percent checked regularly for DS-drug interactions, and two-thirds checked depending on the customers’ health, the type of drug or the type of DS. One-quarter regularly asked about the co-use of prescription drugs (PD) when selling DS, while only 2% asked about the co-use of DS when dispensing PD. Only 25% reported access to independent scientific information on all or most DS sold in their pharmacy. Eight percent had experienced unsafe use of DS by persons with dementia. Six percent had been taught about counselling persons with dementia. Education level influenced professional practice behavior to some extent.

**Conclusion:**

Pharmacy employees do not see themselves as primarily responsible for the safe use of DS by persons with dementia. Moreover, they have limited experience with the unsafe use of DS by these persons. There is potential for improvement regarding tools and educational interventions for pharmacy employees to provide sufficient help to persons with dementia who use DS.

**Electronic supplementary material:**

The online version of this article (10.1186/s12906-019-2587-4) contains supplementary material, which is available to authorized users.

## Background

Dietary supplements (DS) include vitamins, minerals, herbs, amino acids and dietary substances [1]. DS are widely used in the general population [2, 3], often for maintaining or improving health [4]. Considered natural and safe by many, DS can nevertheless compromise patient safety through interactions with prescription drugs (PD) and by causing direct adverse events [5, 6]. Fatal events have been reported [7].

Dementia is a general term for progressive diseases that affect mental abilities and cause problems in activities of daily living (ADL). The majority of persons with dementia have Alzheimer’s disease, with memory problems as the most common symptom [8]. Several DS claim to protect against cognitive decline and dementia, such as omega-3 fatty acids and antioxidants like Vitamin E and Vitamin C, but the scientific evidence is sparse [9, 10]. DS is commonly used by community-dwelling persons with dementia, and studies report a prevalence of 50% [11, 12]. Potentially clinically relevant interactions between DS and PD have been reported in 11–33% of persons with dementia who use DS [6, 12]. Due to reduced cognitive function, persons with dementia are at risk of misdosing or confusing DS with PD or vice versa, imposing additional risks on this particular patient group. Another concern is that two-thirds of persons with dementia using DS receive no help with their DS, even though most of them receive help with administering their PD [12].

General practitioners (GPs) have the responsibility for patients’ health and safety, including their PD [13]. Their responsibility regarding DS is less clear [4]. Patient autonomy is a very strong principle within medical ethics [14], thus patients may freely choose to use DS. However, persons with dementia are often incapable of safeguarding their own use of DS [12]. They may have difficulties in making an informed choice about the use and administration of DS. When patients are not able to take responsibility, who should then be responsible? Should the responsibility rest with the DS retailers who are not a part of the traditional health-care system (hereafter denoted DS retailers), patients’ caregivers, or health-care personnel (e.g., GPs, the home care service or pharmacy employees)? If no one accepts this responsibility, the patient him- or herself will be left responsible. The caregiver might take the initiative for the DS use [12], and can additionally help the patient administer the DS and communicate with the health-care system. Caregivers are an important unpaid care resource [15] but are not expected to have knowledge regarding PD or DS. Furthermore, the DS-retailers should be responsible for giving correct information about the DS content [16], but are not expected to possess knowledge on dementia. We identified GPs, pharmacy employees and the home care service (nurses and nurse assistants) as relevant to this responsibility. These health-care professionals are authorized to work with PD, and the safety of DS use is closely connected to the PD used. We have restricted our research interest to primary health care because it is the backbone of the Norwegian health care system. Furthermore, several patients with late-onset dementia, are diagnosed at the primary health care level and might not have any contact with the specialist health care system.

In Norway, as in several other Western countries, caring for older adults is now in many cases maintained by professional health-care workers, i.e., the home care service, and not by relatives or next of kin. According to Statistics Norway, more than 73% of Norwegian women aged 80 years and older live alone, while 31% of Norwegians (men and women) between 67 and 79 years old live alone [17]. At present, health-care personnel are seldom aware of, or involved in, patients’ DS use [2, 5, 11]. Health-care personnel are obliged to ensure that their patients with cognitive impairments avoid harmful use of PD, but whether health care personnel should also be obliged to take responsibility for the safe use of DS has not, to our knowledge, been addressed previously.

As most pharmacies trade a variety of DS, pharmacy employees are often involved in the sale of DS, [18, 19]. Pharmacists often receive questions about DS from customers, but they neither routinely inquire about DS use, nor monitor or document DS use [20]. Previous publications have revealed room for improvement regarding pharmacists’ knowledge of DS [20, 21]. Most studies regarding pharmacy employees’ experiences with sale/counselling of DS have included only pharmacists as informants. However, employees with other types of educational backgrounds commonly sell DS. We identified only one study in the English language that included pharmacy technicians [22]. Employees with other educational backgrounds than pharmacists account for half of the employees in Norwegian pharmacies [23].

We have previously documented that one-third of persons with dementia recruited from a Norwegian outpatient memory clinic bought their DS in pharmacies [12]. There is a paucity of information about pharmacy employees’ experiences in counselling persons with dementia as part of their daily routine [24]. Even less is known about their experiences counselling persons with dementia or their caregivers about DS. Thus, it is important to explore how pharmacy employees could assist in risk management of DS use in older adults, either by direct counselling or in collaboration with GPs and home care services.

The aim of this study was to describe Norwegian pharmacy employees’ attitudes and professional practice behaviors related to the counselling and sale of DS in general and, more specifically, to persons with dementia. We also investigated to which degree pharmacy employees felt responsible for the safety of customers with dementia buying DS.

## Methods

### Study population and recruitment

**Fig. 1:**
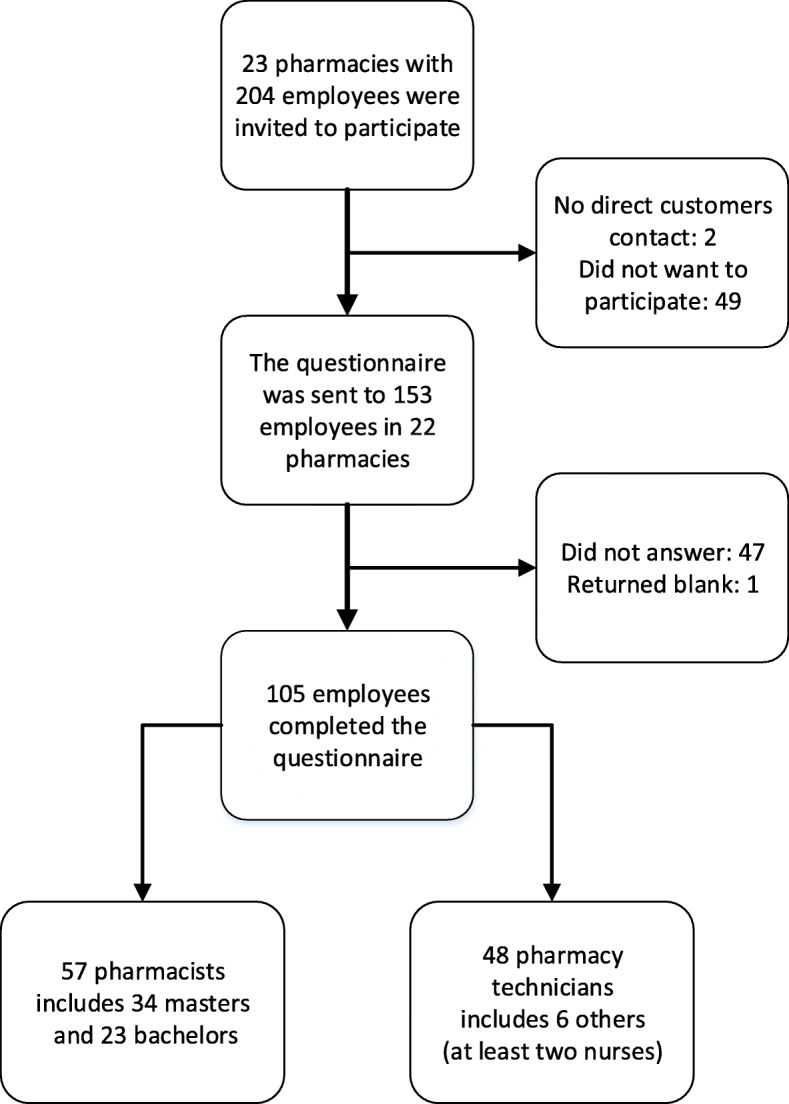
Flowchart of study population and recruitment process

We conducted a cross-sectional survey from December 2014 to March 2015. All pharmacy employees in eight municipalities in Northern Norway were invited to participate. There were 23 pharmacies in this geographical area (one hospital pharmacy and 22 community pharmacies). All but one pharmacy agreed to participate; see Fig. 1 in Result section. The eight municipalities were chosen because they provide the source population for the memory clinic where our research group recently conducted a study of the use of DS by persons with dementia [12]. The present study and further studies among other health-care personnel (GPs and home care service employees) were therefore conducted in the same geographical area.

Employees holding a bachelor’s (three years) or master’s (five years) degree in pharmacy were classified as pharmacists, while employees with other educational backgrounds (e.g., upper secondary school) were classified as pharmacy technicians. We excluded employees without customer contact as part of their daily work. The master’s degree is equivalent to the pharmacist degree across Europe, while the pharmacy bachelor normally qualifies for work as a dispensing pharmacist in community pharmacies. Two of the respondents stated in the open-ended question that they were nurses. Nurses have valuable knowledge that can be used as a resource in pharmacies. In this study, we wanted to ascertain whether there were differences between pharmacists and other employees because we anticipated pharmacists to possess a greater knowledge level regarding adverse events from PD and DS, as well as interactions. Because nurses are not expected to possess the same level of knowledge as pharmacists about these topics, we chose to classify them as pharmacy technicians. In Norway, all health-care personnel are professionals, as they need to apply and receive accreditation from the health authorities. Pharmacy technicians have a vocational qualification after education and practical training and, as front shop staff in the pharmacy, can provide all services, except independently dispensing PD. For instance, they can ask customers about their use of DS. However, they are trained to consult a pharmacist whenever they feel unqualified to solve a problem.

In this study, the home care service is defined as a public well-fare system available to all inhabitants in need, and the backbone of the day-to-day care of community-dwelling people in need of help with medication or ADL-support. When a person needs help with his or her PD, an interdisciplinary collaboration between the pharmacy/pharmacist, GP, and home care service is needed. Pharmacy employees therefore have daily contact with home care service employees and are well acquainted with this service.

Study information and an electronic questionnaire (Questback formula, Questback AS, Oslo, Norway) were sent by e-mail to pharmacy employees. To increase the response rate, we sent three reminder e-mails to all participants. Questback was set up such that respondents could not submit more than one questionnaire. The pharmacy managers informed their employees of the survey and provided the e-mail addresses of all employees who met the inclusion criteria (i.e., in which only employees with customer contact were invited to participate). The number of eligible employees reported by the managers was used to calculate the response rate. Figure 1 provides an overview of the study population and the recruitment process.

### Questionnaire

No validated questionnaire was available that covered our research aspects. Therefore, we designed a questionnaire specifically for this study. As part of this process, we evaluated previous studies and consulted relevant multidisciplinary experts (see acknowledgements). We conducted a feasibility study with fifteen pharmacists and pharmacy technicians to investigate the relevance and readability of the questions and to evaluate the length of the questionnaire. The final version took 10–20 min to complete and covered 35 items, see Additional file 1 for a translated verion of the questionaire. For the present study, we included a subset of items grouped in the following five domains:

1. Study population (gender, education and years of professional experience);
2. Attitudes toward DS (personal DS use, beliefs about positive and negative effects of DS, recommending DS to customers unprompted);
3. Attribution of responsibility for the safety of persons with dementia using DS and suggestions for safety improvement. The employees were asked to rank the following options addressing the question “Where should the responsibility for the safe use of DS by persons with dementia be placed?”: patients themselves, caregivers, GPs, home care services, pharmacies, or DS retailers. DS retailers could be employees in health food stores, internet retailers, complementary and alternative medicine therapists, or others. We also asked the respondents to rank the following suggestions on how to ensure correct and safe use of DS by persons with dementia: information from health authorities to the general population, changes in laws and regulations concerning DS, increased effort from GPs (ask all patients about use of DS and check for adverse events and interactions), increased effort from home care services (convey information about the use of DS to GPs or pharmacists), increased effort from pharmacies (check for interactions between DS and PD for all customers who buy DS, inform GPs when interactions are identified), or DS delivery in multidose drug-dispensing systems together with PD. The multidose drug-dispensing system was not explained further as pharmacy employees are familiar with this system and its implications. The multidose dispensing-system is similar to the Automated Medication Dispensing Systems and is commonly used for PD in Scandinavian countries [25]. A computer-controlled robot system dispenses each patient’s drugs into disposable bags. All drugs intended for one dosing occasion are gathered in one dose unit bag labeled with patient data, drug contents, and the date and time for intake. To deliver DS through the multidose drug-dispensing system, the use of DS would be identified by the health-care system, and both the GP and pharmacist would be responsible for checking for interactions and judgement on safety. It would also facilitate the distribution and administration of DS, thereby avoiding overdoses and other consequences of user error.

The questions about attributed responsibilities and suggestions for improvements of safety were ordinal. Respondents were asked to rank the six categories, resulting in a ranking scale from 1 to 6. We merged priorities 2–4 into a medium-level responsibility category, and priorities 5–6 into a least-responsible category. We were mainly interested in to whom the respondents assigned the most responsibility or what they believed would be the best intervention. For this reason we did not merge priorities 1 and 2. We believe unclear lines of responsibility is an obstacle to the safe care of persons with dementia who use DS. Therefore, we wanted to determine where pharmacy employees placed this responsibility and, additionally, where they placed themselves. In imminent studies that are already designed, we plan to ask caregivers, GPs, and employees in home care service about their opinion on this same matter.

4. Professional practice behaviors related to the sale of DS (questions about DS, where to find independent scientific information about DS, informing customers about potential adverse events from DS, asking about PD use when selling DS and vice versa, checking for interactions between DS and PD (including methods for checking), and willingness to answer questions about DS not bought in the pharmacy)
5. Professional experience with persons with dementia in general and related to DS in particular (education on counselling persons with dementia, experience with persons with dementia who are unable to understand important pharmaceutical information, routines for handling communication problems with customers with dementia, experience with unsafe use of DS among persons with dementia, and routines for handling customers with dementia who use DS unsafely).

Five of the questions in the subset used in this study were open-ended questions (which DS products they believe have positive effects against dementia, routines for handling communication problems, routines for handling unsafe DS use by customers with dementia, in which context they have received education on counselling persons with dementia, and methods for checking for interactions between DS and PD), two were ordinal (attributed responsibility, suggestions for improvement of safety), and the remainder were dichotomous or multiple-choice questions. It was not possible to add free text in the questionnaire except for the open-ended questions.

### Ethics

The Regional Committee for Medical and Health Research Ethics presented no objections to the study design (2014/1385). As no patients were included, the project was defined as “quality assurance”. The survey did not collect personally identifiable information and therefore was not accountable to the Norwegian Data Protection Agency. All participants were given written information about the study and informed that submitting the questionnaire was considered to be study consent.

### Statistics

We used IBM SPSS (Statistical Package for the Social Sciences) version 23.0 (IBM Corp., Armonk, NY, US) for the statistical analyses. Data are presented as absolute and relative frequencies. We used Pearson’s chi-square test or Fisher’s exact test for categorical data. *P* values *< 0.05* were considered statistically significant.

## Results

### Study population

One hundred and five persons, 11% men (*n* = 12) and 89% women (*n* = 93), answered the questionnaire, resulting in a response rate of 52%. Of these respondents, 54% were pharmacists, and 46% were pharmacy technicians, see Fig. 1. Four percent had 0–1 year (*n* = 4), 27% had 1–5 years (*n* = 28), 37% had 6–15 years (*n* = 39), and 32% had more than 16 years (*n* = 34) of professional experience.

### Attitudes toward DS

In total, 37% of the respondents (n = 39) reported that they currently used or had previously used some type of DS. Nine percent (*n* = 9) believed that some DS might have symptomatic or prophylactic effects against dementia. The following DS were reported to be effective by the 10 respondents who answered this open-ended question (descending order of frequency): omega-3-fatty acids, *Ginkgo biloba*, folic acid, vitamin E, vitamin B, vitamin C, flavonoids, lecithin, cranberries, garlic and ginger. Fifty-nine percent of the respondents (*n* = 62) agreed that DS could have potentially harmful effects on users’ health, and more pharmacists than pharmacy technicians agreed with this statement (Table 1). There were no other differences between the pharmacists and pharmacy technicians in attitudes toward DS.

**Table 1:** Pharmacists’ and pharmacy technicians’ attitudes toward dietary supplements

	Pharmacist		Pharmacy technicians		Total		*p*-value^a^
	*n* = 57		*n* = 48		*n* = 105		
	n	%	n	%	n	%	
Personal use of DS (past or current)^b^	25	44	14	29	39	37	0.131
Believe DS have effects against dementia^b, c^	5	9	4	8	9	9	0.172
Agrees that DS can cause harm to health^b, c^	44	77	18	38	62	59	**< 0.001**
Recommend DS to customers unprompted^d^	21	37	16	33	37	35	0.369

*DS* Dietary supplements. Pharmacists include employees with a bachelor’s or master’s degree in pharmacy. Pharmacy technicians include employees with other educational backgrounds, mainly pharmacy technicians (upper secondary school). ^a^Fisher’s exact test; ^b^Data are missing for one employee. ^c^The categories tested were yes, no and do not know. ^d^Data are missing for two persons. Significant comparisons are printed in bold

Pharmacy employees who used DS themselves more often recommended DS to customers unprompted (54% of users (*n* = 21) vs. 23% of nonusers (*n* = 15), *p* = 0.002). Of the 35% (*n* = 37) who recommended DS unprompted to customers, 73% (*n* = 27) did so because they believed that DS have documented beneficial effects, and 49% (*n* = 18) because they believed that DS could cure or give symptomatic relief. Twenty-seven percent (*n* = 10) recommended DS because they felt the customers wanted to buy DS, 16% (*n* = 6) due to the pharmacy’s upselling policy and 3% (n = 1) because they believed that the products did no harm. Of the 63% (*n* = 66) who never recommended DS unprompted, 62% (*n* = 41) reported insufficient knowledge of DS, 62% (n = 41) feared interactions with PD, 42% (*n* = 28) feared adverse events, and 20% (*n* = 13) did not believe DS to have positive effects (it was possible to choose more than one reason). Two respondents did not answer the question about whether they recommended DS.

### Attributed responsibility

**Fig. 2:**
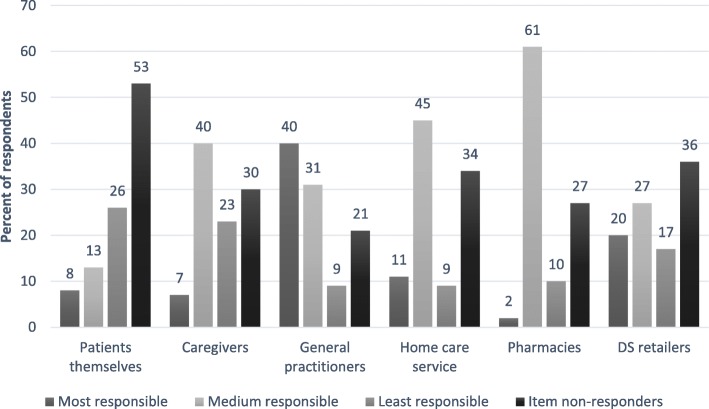
The employees’ ranking of responsibility for the safety of persons with dementia who use dietary supplements. The employees were given six options addressing the question “Where should the responsibility for the safe use of DS by persons with dementia be placed?” Respondents were asked to rank the six categories, resulting in a ranking scale from 1 to 6. We merged priorities 2–4 into a medium-level responsibility category and priorities 5–6 into a least-responsible category. DS retailers could be employees in health food stores, internet merchandisers, complementary and alternative medicine therapists, or others

When asked to rank the six options, the majority of the respondents stated that GPs should be responsible for ensuring the safe use of DS by persons with dementia (Fig. 2). They assigned themselves a medium level of responsibility, followed by home care services and caregivers. Only 2% (n = 2) indicated that pharmacies should bear the greatest responsibility. Patients themselves and their caregivers were considered to bear the least responsibility.

Sixty-two percent (*n* = 65) of the pharmacy employees chose GPs, while 36% (*n* = 38) chose pharmacies when answering the following question: “Do you think GPs or pharmacies should be responsible for routinely checking for interactions between DS and PD in persons with dementia who use DS?” Two respondents did not answer this question.

**Fig. 3:**
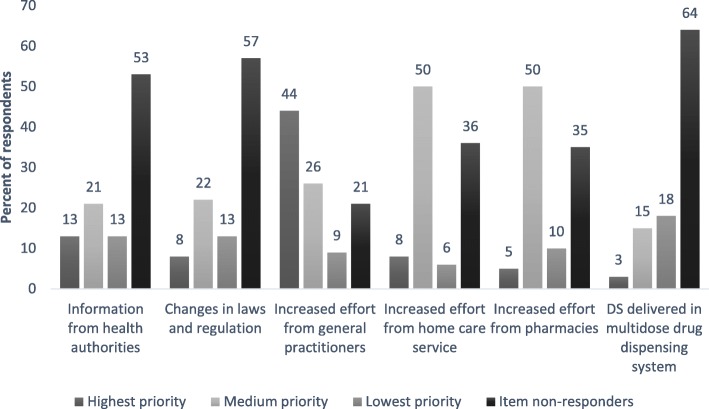
Pharmacy employees’ opinions on how to improve the safety of persons with dementia who use dietary supplements. DS, dietary supplements. The employees were given six alternatives on how to ensure the correct and safe use of DS. Respondents were asked to rank the six categories resulting in a ranking scale from 1 to 6. We merged priorities 2–4 into a medium-level priority category and priorities 5–6 into a lowest-priority category

Most employees gave the highest priority to increased effort from GPs when asked to rank several options addressing the question “Which option is best to ensure the correct and safe use of DS by persons with dementia?” (Fig. 3). Increased effort from home care services and pharmacies were ranked approximately equally at medium priority.

### Professional practice behaviors

#### Questions about DS and the availability of independent scientific information on DS

Most employees (96%, *n* = 101) had received questions about DS from customers in the pharmacy, including 11% (*n* = 11) daily and 39% (*n* = 41) weekly. Two of the respondents did not answer this question. There were no differences between pharmacists and pharmacy technicians. Eighty-one percent (*n* = 85) had been asked to provide information about DS-products not sold in their pharmacy. One respondents did not answer this question. More pharmacists than pharmacy technicians received questions about DS not sold in the pharmacy (95% against 65%, *p* < 0.001). Twenty-seven percent (*n* = 28) would answer the question about DS not sold in the pharmacies. Eleven of the respondents did not answer this question. More pharmacists than pharmacy technicians provided information about DS not sold in their pharmacy (χ ^2^ = 10.784 (2), *p* = 0.005)^*^. Five percent of respondents reported access to independent scientific information on all DS sold in their pharmacy, 20% had information on most products, and 30% had information on a few products. Forty-two percent did not have such information available. Three of the respondents did not answer this question. There was no difference between pharmacists and pharmacy technicians.

#### General safety procedures related to DS

Ninety percent of the pharmacy employees provided information on possible adverse events, including interactions (48% confirmed that they informed customers about potential adverse events, including interactions; 42% indicated that they did this sometimes). Additionally, 82% checked for interactions at least sometimes, while 16% did this as a routine (Table 2). More pharmacists than pharmacy technicians provided information on possible adverse events and asked about the co-use of DS and PD, but there was no difference in checking for interactions. A minority of the respondents (14 pharmacy technicians and 25 pharmacists) answered the question about which sources they used to check for interactions. Among pharmacy technicians, five asked pharmacists to perform the interaction analysis, five analyzed it themselves with the help of various Norwegian databases, and four sometimes checked themselves and sometimes asked a pharmacist to check. Most pharmacists used various internet sources, mainly webpages organized by official health authorities, e.g., relis.no (the official web page of the Norwegian Pharmacovigilance centers. These centers are run by Norwegian Health Authorities. Relis.no answers questions about adverse events and interactions from PD and DS). One pharmacists used only the product-dependent medication information leaflet. We found no difference in safety procedures depending on the employees’ attitudes toward DS. Believing that DS have no negative effects was not associated with less thorough safety routines, specifically, performing interaction analyses (*p* = 0.328), asking about the co-use of DS when dispensing PD (*p* = 0.374), asking about the co-use of PD when selling DS (χ^2^ = 3.86(6), *p* = 0.526)^*^ and informing about adverse events including interactions (*p* = 0.344). Statistics were performed using Fisher’s exact test and chi square test^*^. The same categories were tested as in Table 2 (that is, yes, no, and sometimes).

**Table 2:** Pharmacy employees’ professional practice behaviors related to dietary supplements

	Pharmacists		Pharmacy technicians		Total		*p*-value
	n = 57		n = 48		n = 105		
	n	%	n	%	n	%	
Give information on adverse events and possible interactions^a, b^							**0.022**
Yes	30	53	21	44	51	48	
Sometimes	26	46	18	38	44	42	
No	1	2	8	17	9	9	
Ask customers about PD use when selling DS^a, b^							**0.021**
Always	15	26	11	23	26	25	
Sometimes^c^	41	72	28	58	69	66	
Depending on the customers’ health	9	16	0	0	9	9	
Depending on the type of DS	32	56	28	58	60	57	
Never	1	2	8	17	9	8	
Ask customers about DS use when dispensing PD^a, d^							**0.002**
Always	0	0	2	4	2	2	
Sometimes	32	56	12	25	44	42	
Never	24	42	33	69	57	54	
Routinely check for interactions between DS and PD^e, f^							0.409
Always	7	12	10	21	17	16	
Sometimes^f^	40	70	28	58	68	65	
In certain patients groups	12	21	3	6	15	14	
For certain DS	34	60	18	38	52	50	
For certain PD	16	28	17	35	33	31	
Never	10	18	7	15	17	16	

*DS* dietary supplements, *PD* prescription drugs

Pharmacists include employees with bachelor’s or master’s degree in pharmacy. Pharmacy technicians include employees with other educational backgrounds, mainly pharmacy technicians (upper secondary school)

^a^Statistics are from Fisher’s exact test. The answers always, sometimes and never are included

^b^Data for one employee are missing

^c^Respondents who confirmed that they asked customers about PD when selling *DS* depending on the customers’ health or depending on the type of DS were merged into the category “Sometimes”

^d^ Data for two employees are missing

e Data for three employees are missing

^f^Statistics are from the chi-square test

^g^It was possible to give more than one answer to this question. Respondents who confirmed that they checked for interactions in one or more of the following cases: certain patient groups, for certain DS or for certain PD, were merged into the category “Sometimes”

Significant comparisons are printed in bold

#### Professional experience with customers with dementia, both in general and related to DS

Fifty-three percent (*n* = 56) had experienced customers who were unable to understand important pharmaceutical information because of cognitive problems; 26% (*n* = 27) were uncertain, while 21% (*n* = 22) had never been in this situation. More pharmacists than pharmacy technicians had experienced this situation (χ ^2^ = 9.685(2), *p* = 0.008)*. The open-ended question about routines for handling communication problems was answered by 54 respondents, of which 31 reported that their pharmacy lacked routines to handle this type of communication problem. The respondents mentioned the following three interventions (in decreasing frequency): making contact with the caregiver/GP/home care service, oral or written information given to the person with dementia, and taking the initiative for the client/person with dementia to have his or her medication dispensed by the home care service in the multi-drug-dispensing system (mentioned only by two respondents). Ten of the 31 respondents who stated that their pharmacy lacked a routine, mentioned interventions that had taken place in their pharmacy. Six percent of the employees had received education on counselling persons with dementia. Three respondents did not answer this question. When asked in which context the respondents had received dementia education, only six respondents who had received such education answered this question, two of whom had received this training during nursing education and one during five years of employment at a dementia department of a nursing home. The rest stated that they received this education during their professional education without further specification.

Eight percent (*n* = 8) of the employees (seven pharmacists and one pharmacy technicians) had experienced persons with dementia with unsafe use of DS. When asked “How do you act professionally when you discover such a problem”, only five respondents answered the question, one contacted the caregivers, and the others tried to inform the persons with dementia more thoroughly about the hazards and encouraged them to contact their GPs. Statistics were performed using chi square test*.

## Discussion

### Interpretation of the results

This study revealed that the pharmacy employees had a rather conservative attitude toward the use of DS. A minority of the respondents (9%) believed DS to be effective against dementia; a majority (59%) agreed that DS could cause harm. One-third of the respondents (37%) were DS users themselves. We have not found other studies addressing the question of the effectiveness of DS against dementia, but the proportion of pharmacists who agree with the general statement that most DS/herbs/natural products are clinically effective has been found to vary from 19 to 48% [20, 26]. Our results regarding perceived health risks are in line with those of a systematic review reporting that 50% of pharmacists believed DS to have potentially harmful effects [20]. Interestingly, even though some employees did not think that DS could have negative effects, this attitude did not seem to influence their counselling or safety procedures.

Previous studies have reported past or current DS use by 53–66% of pharmacists [26–28]. In line with the results of other studies, our results confirm that personnel who use DS themselves more often recommend DS to their customers [26, 27] possibly due to a generally more positive attitude toward DS among self-users [27]. Other studies have suggested that 40–91% of pharmacists recommend DS to customers [20, 26, 27]. These studies do not specify whether the recommendations were unprompted or resulted from customers’ requests, except in one study where 38% of the pharmacists recommended DS unprompted [26], which is comparable to our findings (35%). The most common reason for recommending DS among our respondents was an assumed documented effect. The most common reasons for not recommending were lack of knowledge of DS and fear of interactions between DS and PD, in line with the results of a previous qualitative study [29]. We find it reassuring that upselling was an uncommon reason for recommending DS.

Studies have identified potentially clinically relevant interactions between DS and PD in persons with dementia [6, 12]. Together with a lack of help with DS administration [12], these interactions indicates that at least some persons with dementia might be exposed to health risk (e.g., overdose and adverse effects) because of their DS use. Previous studies have highlighted the need for pharmacists to routinely document, monitor and inquire about customers’ use of DS [20]. Our results showed that pharmacy employees perceive themselves and home care service employees as contributors to safeguarding the use of DS by persons with dementia, but pharmacy employees suggest that GPs should be the main responsible care-taker in maintaining patient safety. Eight percent had experienced unsafe use of DS among customers with dementia. This finding might indicate that unsafe use is an infrequent problem, or that pharmacy employees do not possess the means necessary to identify such problems. Necessary means would include knowledge of dementia and resources for proper counselling, including routines for identifying problematic use.

First, pharmacy employees might have limited knowledge of dementia. Studies investigating general knowledge of dementia among pharmacists and final year pharmacy students have indicated potential for improvements in knowledge of risk factors, caregiving issues and the pharmacological management of Alzheimer’s disease [30]. We did not measure the level of knowledge of dementia as such but did notice that the respondents stated that they had not been educated on counselling persons with dementia.

Second, our study suggests a lack of resources and routines for counselling persons with dementia on their use of DS. Norwegian pharmacies do not have access to medical records, which limits the possibility of identifying users’ dementia disease. Even if employees are aware of customers’ cognitive problems, they are unlikely to have knowledge on DS use and the conditions of such use unless they specifically ask about it. As the majority of persons with dementia buy their DS from health food shops or on the internet [12], it is difficult to intercept their DS use at the pharmacy. Additionally, as impaired insight is a common feature of dementia, the persons themselves might say the use is unproblematic when in fact it is not. Not being fully informed about DS use makes interaction analyses uncertain. Our results suggest shortcomings in employees’ safety routines, as only 2% routinely asked about the co-use of DS when dispensing PD and only a minority would provide information about DS bought outside the pharmacy. Moreover, as shown by others, pharmacy employees lack independent scientific information on most of the DS sold in their pharmacies [26], which may influence their motivation to take on the responsibility [19]. Formulating legislation to clarify the legal and professional role of pharmacists with respect to DS could make it easier to take on the responsibility [31].

Our results suggest that pharmacy technicians have less stringent procedures than pharmacists, as they inquire about the co-use of DS and PD less often. They also inform patients less often about side effects and about DS-products not sold in the pharmacy. This fact is important, as pharmacy technicians receive questions about DS as often as pharmacists. Persons with dementia and their caregivers are more likely to purchase DS from a pharmacy technician than from a pharmacist because there are as many pharmacy technicians as pharmacists in Norwegian pharmacies, and pharmacy technicians sell more over-the-counter products while pharmacists work more with prescriptions. The difference in professional practice behaviors between pharmacists and pharmacy technicians demonstrated here and in one other study [22] might be explained by higher knowledge level regarding PD and DS among pharmacists due to different education levels. It could also reflect a more strongly perceived professional responsibility among pharmacists to give evidence-based advice [32]. Only a minority of pharmacy technicians agreed with the statement that DS can cause harm to health. We have not, as mentioned earlier, shown any connection between this belief and the presence of less strict safety routines.

Compared with increased effort from health personnel, the remaining measures suggested to ensure safe use of DS by persons with dementia were less popular. These included changes in laws and regulations, information from health authorities and dispensing DS in a multidose drug-dispensing system (drug-dispensing system). Few respondents were positive toward including DS in a drug-dispensing system. One explanation could be that the employees do not consider the reconciliation of DS to be feasible, either due to a lack of studies testing DS safety [33], lack of independent scientific information [34], or other reasons. In addition, some products, such as transparent tablets, large tablets, oral lyophilisates, tablets with a short shelf-life and tablets that cannot be stored at room temperature, are excluded from the drug-dispensing system because of technical limitations (Annette Vik Jøsendal, Apotek 1, personal communication). However, this is true for PD as well as DS.

As mentioned earlier, only a minority of pharmacy employees considered actions outside the primary health-care system to be important. Changes in laws and regulations could enforce control over the content of DS products and regulate both DS retailers’ and health-care personnel’s professional conduct more thoroughly, including the provision of clear lines of responsibility. However, enforcement would also require increased resources. Information campaigns from health authorities might be less effective due to difficulties in reaching persons with dementia.

### Methodological considerations

We included pharmacy employees using minimal exclusion criteria to maintain external validity. The response rate was adequate compared with those in similar studies [20], but the limited number of respondents may weaken the study power and generalizability. The study population is representative of the Norwegian setting in terms of the gender distribution [23]. However, employees with a pharmacy degree were overrepresented. Our study population comprised 32% master’s degrees, 22% bachelor’s degrees, and 46% technicians (including others), while the national distribution is (by Des 2017) 25% master’s degrees, 19% bachelor’s degrees, and 56% pharmacy technicians (including others) [23]. Our demographic findings are comparable to those of other surveys among Norwegian pharmacy employees performed in different geographical areas [35, 36]. Considering this potential limitation, the study findings may be generalizable to pharmacy employees in other countries with similar health-care systems, pharmacies and education programs, particularly Swedish and Finnish pharmacies, which also employ bachelor pharmacists [37]. Few studies have evaluated the use of DS by the Norwegian general population, but available data indicate higher use among Scandinavian women than in women from southern Europe [38]. A report from the Norwegian Food Authority found that 500 Norwegian respondents use an average of 3.7 DS. As an average for the Nordic countries, the respondents used 3.6 different products [39]. We believe, however, that the question regarding pharmacy employees’ professional conduct and responsibility toward customers with dementia who buy DS is relevant from a global perspective.

Even though we provided written information stating how we defined DS of interest, we cannot determine if the definition was clear to all employees. Similarly, we do not know if all employees shared a common interpretation of the word dementia, since we did not give a specific definition.

Few respondents answered the question on how the safety of persons with dementia who use DS could be improved. The reason could be that they disagreed with the need for improvement, and this answer should have been included as a possible response. Another reason could be that they found the ranking difficult. Further specification of the options “Information from health authorities to the general population”, and “Changes in laws and regulations concerning DS” should also have been provided for clarity, such as “Information from health authorities to the general population about DS” and "Changes in laws and regulations concerning DS (indicates increased control of the DS content, such as increased testing for toxic effects, because DS currently has less strict safety routines than PD). Further specification might have increased the response rate to these questions and increased the certainty of the respondents about their answers. Regarding the option “increased effort from the home care service”, we could also have been more specific. A suggested specification regarding this question could have been, “If the home care service discovers unsafe use of DS by persons with dementia, they should convey this information to the GP or pharmacy”. The question about work length, used to describe the study-population, had wording that might have led to uncertainty among respondents who had worked exactly 16 years. The response rate to this question was 100%, so this uncertainty did not stop respondents from answering. Twenty-nine percent of the pharmacy technicians answered that they routinely informed patients/customers about DS when dispensing PD. This question should have been posed to pharmacists only, as both dispensing PD and asking about co-use of other products are the pharmacist’s duties after the technician has prepared the prescription. Based on our experience from pharmacy practice, we believe that communication about co-use would have taken place in close cooperation with a pharmacist. We also think the proportion who answered yes was very high in both groups; two technicians even said they always asked, which suggests some level of “eager to please” bias. The question “Do you supply information on adverse events from DS including possible interactions?” was provided with the options yes, no, and sometimes. A further specification concerning when to answer yes or sometimes was not provided, which could have led to inconsistent responses. However, when the option yes is provided as a different response to sometimes, it implies a regular intervention. The response rate was 99% to this question, as one respondent did not answer. The main difference between pharmacists and pharmacy technicians was that more pharmacy technicians answered no to this question.

The study design made it necessary for the participants to answer electronically. This could have induced some obstacles to participation; however, it was possible to answer by using the pharmacy’s computer, so it was not necessary to possess a personal computer to participate.

### Implications and future research

Currently, there is a focus on dementia-friendly pharmacies and the special needs of persons with cognitive problems [40]. We recommend clearly defined routines to handle communication problems and to make existing routines known to all employees. The focus on safe sale of DS in general should be improved. Frequent assessments of potential interactions and routine inquiries about DS, whether or not they are bought in the pharmacy, are needed, as well as adequate independent scientific information on all products sold in the pharmacy. If pharmacies were to initiate actions to improve these measures, a longitudinal study of the effect of such an intervention would be recommended.

We believe that multidisciplinary collaboration among pharmacists, GPs, home care service employees and caregivers can ensure the safe use of DS by persons who are incapable of handling the use themselves due to dementia. First, the use needs to be identified. Second, it is important to assess interactions between DS and PD and potential adverse reactions. Third, the team needs to consider the health benefits of using DS and recommend use or discontinuation. If use is to be continued, it is important to plan for safe administration, for instance administration by the home care service. In all steps, the involvement of the patients or caregivers is recommended.

## Conclusion

The pharmacy employees showed a conservative attitude toward DS in general and had limited experience with problematic DS use among persons with dementia. They did not rate themselves as primarily responsible for the safety of persons with dementia who use DS. Contributing factors to this view may be the lack of independent scientific information on DS product, limited information on customers’ medical conditions and limited knowledge on how to communicate with persons with dementia. The roles and responsibilities concerning the safety of persons with dementia using DS need to be clearly defined. We suggest collaborations between the pharmacy and the GP that preferably include home care services and caregivers.

## Additional file

Additional files 1: Questionnaire. Translated questionnaire answered by the respondents. (DOCX 52 kb)

## Data Availability

The datasets used and/or analyzed during the current study are available from the corresponding author upon reasonable request.

## Abbreviations

ADL: Activities of daily living
DS: Dietary supplement
GP: General practitioner
PD: Prescription drug

## References

[1] Services USDoHH. Dietary Supplement Health and Education Act of 1994. Public Law No. 103-417.Paragraph 3a.1994. https://ods.od.nih.gov/About/DSHEA_Wording.aspx.

[2] Djuv A, Nilsen OG, Steinsbekk A (2013). The co-use of conventional drugs and herbs among patients in Norwegian general practice: a cross-sectional study. BMC Complement Altern Med.

[3] Garcia-Alvarez A, Egan B, de Klein S, Dima L, Maggi FM, Isoniemi M, Ribas-Barba L, Raats MM, Meissner EM, Badea M (2014). Usage of plant food supplements across six European countries: findings from the PlantLIBRA consumer survey. PLoS One.

[4] Starr RR (2015). Too little, too late: ineffective regulation of dietary supplements in the United States. Am J Public Health.

[5] Bello N, Winit-Watjana W, Baqir W, McGarry K (2012). Disclosure and adverse effects of complementary and alternative medicine used by hospitalized patients in the north east of England. Pharm Pract (Granada).

[6] Dergal JM, Gold JL, Laxer DA, Lee MS, Binns MA, Lanctot KL, Freedman M, Rochon PA (2002). Potential interactions between herbal medicines and conventional drug therapies used by older adults attending a memory clinic. Drugs Aging.

[7] Kupiec T, Raj V (2005). Fatal seizures due to potential herb-drug interactions with Ginkgo biloba. J Anal Toxicol.

[8] Winblad B, Amouyel P, Andrieu S, Ballard C, Brayne C, Brodaty H, Cedazo-Minguez A, Dubois B, Edvardsson D, Feldman H (2016). Defeating Alzheimer's disease and other dementias: a priority for European science and society. Lancet Neurol.

[9] Persson T, Popescu BO, Cedazo-Minguez A (2014). Oxidative stress in Alzheimer's disease: why did antioxidant therapy fail. Oxidative Med Cell Longev.

[10] Dangour AD, Andreeva VA, Sydenham E, Uauy R (2012). Omega 3 fatty acids and cognitive health in older people. Br J Nutr.

[11] Landin J, Frolich L, Schwarz S (2008). Use of alternative therapies in patients with dementia and mild cognitive impairment: a prospective, controlled study. Int J Geriatr Psychiatry.

[12] Risvoll H, Giverhaug T, Halvorsen KH, Waaseth M, Musial F (2017). Direct and indirect risk associated with the use of dietary supplements among persons with dementia in a Norwegian memory clinic. BMC Complement Altern Med.

[13] Edersheim JG, Stern TA (2009). Liability associated with prescribing medications. Prim Care Companion J Clin Psychiatry.

[14] Beauchamp TL, Childress J. Principles of biomedical ethichs. 6th edn: Oxford University Press; 2009.

[15] Handels Ron L.H., Sköldunger Anders, Bieber Anja, Edwards Rhiannon Tudor, Gonçalves-Pereira Manuel, Hopper Louise, Irving Kate, Jelley Hannah, Kerpershoek Liselot, Marques Maria J., Meyer Gabriele, Michelet Mona, Portolani Elisa, Røsvik Janne, Selbaek Geir, Stephan Astrid, de Vugt Marjolein, Wolfs Claire, Woods Bob, Zanetti Orazio, Verhey Frans, Wimo Anders (2018). Quality of Life, Care Resource Use, and Costs of Dementia in 8 European Countries in a Cross-Sectional Cohort of the Actifcare Study. Journal of Alzheimer's Disease.

[16] Coon SA, Stevens VW, Brown JE, Wolff SE, Wrobel MJ (2015). Comparison of dietary supplement product knowledge and confidence between pharmacists and health food store employees. J Am Pharm Assoc (2003).

[17] Mørk E. Seniorer i Norge 2010/Seniors in Norway 2010 (Report in Norwegian with English abstract). Statistics Norway. 2011. https://www.ssb.no/sosiale-forhold-og-kriminalitet/artikler-og-publikasjoner/_attachment/157022?_ts=14370df7000. Accessed:21 Nov 2018.

[18] Waaseth M, Eggen AE, Grimsgaard S (2007). Natural remedies in Scandinavia-authorization and sales. Pharm World Sci.

[19] Boon H, Hirschkorn K, Griener G, Cali M (2009). The ethics of dietary supplements and natural health products in pharmacy practice: a systematic documentary analysis. Int J Pharm Pract.

[20] Kwan D, Hirschkorn K, Boon H (2006). U.S. and Canadian pharmacists' attitudes, knowledge, and professional practice behaviors toward dietary supplements: a systematic review. BMC Complement Altern Med.

[21] Waddington F, Naunton M, Kyle G, Thomas J, Cooper G, Waddington A (2015). A systematic review of community pharmacist therapeutic knowledge of dietary supplements. Int J Clin Pharm.

[22] Jordan MA, Foster K, Gandhi A, Mohebbi N, Tehrani L (2011). Assessment of herbal weight loss supplement counseling provided to patients by pharmacists and nonpharmacists in community settings. J Am Pharm Assoc.

[23] Trained healthcare professionals. Key figures: Pharmacies and pharmaceuticals in Norway. The Norwegian Pharmacy Union. The Norwegian Pharmacy Union (Apotekerforeningen). 2018. [https://www.apotek.no/in-english/education]. Accessed:10 Sept 2018.

[24] Barry HE, Parsons C, Passmore P, Hughes CM (2013). Community pharmacists and people with dementia: a cross-sectional survey exploring experiences, attitudes, and knowledge of pain and its management. Int J Geriatr Psychiatry.

[25] Johnell K, Fastbom J (2008). Multi-dose drug dispensing and inappropriate drug use: a nationwide register-based study of over 700 000 elderly. Scand J Prim Health Care.

[26] Welna EM, Hadsall RS, Schommer JC (2003). Pharmacists’ personal use, professional practice behaviors, and perceptions regarding herbal and other natural products. J Am Pharm Assoc.

[27] Howard N, Tsourounis C, Kapusnik-Uner J (2001). Dietary supplement survey of pharmacists: personal and professional practices. J Altern Complement Med.

[28] Gardiner P, Woods C, Kemper KJ (2006). Dietary supplement use among health care professionals enrolled in an online curriculum on herbs and dietary supplements. BMC Complement Altern Med.

[29] Culverhouse SE, Wohlmuth H (2012). Factors affecting pharmacists' recommendation of complementary medicines - a qualitative pilot study of Australian pharmacists. BMC Complement Altern Med.

[30] Zerafa N, Scerri C (2016). Knowledge and pharmacological management of Alzheimer's disease by managing community pharmacists: a nationwide study. Int J Clin Pharm.

[31] Song M, Ung CO, Lee VW, Hu Y, Zhao J, Li P, Hu H (2017). Community pharmacists’ perceptions about pharmaceutical service of over-the-counter traditional Chinese medicine: a survey study in Harbin of China. BMC Complement Altern Med.

[32] Braund R, Chesney KM, Keast EP, Ng LJ, Qi S, Samaranayaka S, Wang E (2012). Are all pharmacy staff interested in potential future roles?. Int J Pharm Pract.

[33] Shaw D, Ladds G, Duez P, Williamson E, Chan K (2012). Pharmacovigilance of herbal medicine. J Ethnopharmacol.

[34] Raynor DK, Dickinson R, Knapp P, Long AF, Nicolson DJ (2011). Buyer beware? Does the information provided with herbal products available over the counter enable safe use?. BMC Med.

[35] Driesenaar JA, De Smet PA, van Hulten R, Horne R, Zwikker H, van den Bemt B, van Dulmen S (2016). Beliefs about inhaled corticosteroids: comparison of community pharmacists, pharmacy technicians and patients with asthma. J Asthma.

[36] Morken T, Fossum S, Horn AM, Granas AG (2008). Self-efficacy in counseling in Norwegian chain pharmacies: a cross-sectional study. Res Social Adm Pharm.

[37] Ansatte i apotek i Norden/Employees in pharmacies in the Nordic countries (web page in Norwegian). The Norwegian pharmacy Union (Apotekerforeningen). The Norwegian pharmacy Union (Apotekerforeningen). 2018. [https://www.apotek.no/Files/Filer_2014/Engelske_sider/Pharmacies%20and%20prescriptions%20-%20%20Key%20Figures%202017.pdf]. Accessed 10 Sept 2018.

[38] Skeie G, Braaten T, Hjartaker A, Lentjes M, Amiano P, Jakszyn P, Pala V, Palanca A, Niekerk EM, Verhagen H (2009). Use of dietary supplements in the European prospective investigation into Cancer and nutrition calibration study. Eur J Clin Nutr.

[39] Danielsen S**,** Ekrol PS**.** Food supplements in the Nordic countries. The Norwegian Food Authorities. 2009. https://www.mattilsynet.no/mat_og_vann/spesialmat_og_kosttilskudd/kosttilskudd/food_supplements_in_the_nordic_countries__results_from_survey_among_consumers.4137/binary/Food%20supplements%20in%20the%20Nordic%20countries%20-%20results%20from%20survey%20among%20consumers. Accessed: 11.11.18.

[40] Gilmartin-Thomas JF, Orlu M, Alsaeed D, Donovan B. Using public engagement and consultation to inform the development of ageing- and dementia-friendly pharmacies - innovative practice. Dementia (London). 2017:1471301217725896. 10.1177/1471301217725896. 10.1177/1471301217725896 28854813

