# Supplementary material for: Pharmacy employees’ involvement in safeguarding persons with dementia who use dietary supplements: Results from a survey of Norwegian pharmacies
Source: BMC Complement Altern Med. 2019 Jul 19;19:179. doi: 10.1186/s12906-019-2587-4 (PMC6642513; doi:10.1186/s12906-019-2587-4)
Supplement: Supplementary file 1 — Questionnaire. Translated questionnaire answered by the respondents. (DOCX 52 kb) [file 12906_2019_2587_MOESM1_ESM.docx]

Questionnaire

Translated version, full questionnaire. Questions included in the manuscript are marked by asterisk (*)

*

1. Gender?
   1. Male
   2. Female

*

1. How long have you worked in a pharmacy?
   1. Less than one year
   2. 1-5 years
   3. 6-15 years
   4. More than 16 years

*

1. What is your educational background?
   1. Pharmacist, master’s degree
   2. Pharmacist, bachelor’s degree
   3. Pharmacy technicians
   4. Other

*

1. Do you believe some DS might have effect against dementia (symptomatic or prophylactic)?
   1. Yes
   2. No
   3. I do not know

*

1. If yes; which DS products do you believe might have effect?

Open-ended question

*

1. Do you currently use DS or have you used DS in the past?
   1. Yes
   2. No
2. If yes; which DS do you use, or have you used in the past?

Open-ended question

*

1. Have you experienced pharmacy customers who do not understand important pharmaceutical information due to dementia?
   1. Yes
   2. No
   3. Uncertain

*

1. If yes, does your pharmacy have routines or a common practice to handle this? Please, specify.

Open-ended question

*

1. Are you aware of pharmacy customers whom you fear have unsafe use of DS due to dementia?
   1. Yes
   2. No

*

1. If yes; how do you act professionally when you discover such a problem?

Open-ended question

1. Have you received education on counselling with persons with dementia?

*

- 1. Yes
  2. No

1. If yes, specify in which context you received this education

*

Open-ended question

1. Where should the responsibility for the safe use of DS by persons with dementia be placed? It is possible to choose more than one answer. Prioritize the alternatives.

*

- 1. The person with dementia him/her-self
  2. The caregivers
  3. The DS retailer(health food stores, internet merchandisers or complementary and alternative medicine therapists et cetera)
  4. The pharmacy
  5. The GP
  6. Home care service

*

1. Do you think GPs or pharmacists should be responsible for routinely checking for DS-PD interactions in persons with dementia who use DS?
   1. GPs
   2. Pharmacists
2. How often do you receive questions about DS?

*

- 1. Daily
  2. Weekly
  3. Monthly
  4. Less often than monthly
  5. Never

*

1. Are you asked to provide information about DS-products not sold in your pharmacy?
   1. Yes
   2. No

*If the answer is no, the respondents should go directly to Question 19.*

*

1. If yes, do you provide information on DS-products not sold at your pharmacy?
   1. Yes
   2. No
2. Does yours pharmacy upsell DS as a routine?
   1. Yes
   2. No

*

1. Does your pharmacy check for PD-DS interactions as a routine when selling DS? It is possible to choose more than one alternative.
   1. Yes, always
   2. Yes, but only for certain DS
   3. Yes, but only for certain PD
   4. Yes, but only in certain patients groups
   5. No
2. If a customer wants to buy DS, do ask about intended use?
   1. Yes
   2. No
   3. Sometimes

*

1. Do you routinely ask customers about DS use when dispensing PD?
   1. Yes
   2. In certain cases
   3. No

*

1. Do you sometimes recommend DS to customers unprompted?
   1. Yes
   2. No

*If the answer is yes, the respondent should continue to Question 24 and skip Question 25. If the answer is no, the respondents should go directly to Question 25.*

*

1. If yes, which criteria are your recommendation based on? (It is possible to choose more than one reason).
   1. The DS has a documented beneficial effect
   2. A belief that DS would cure or give symptomatic relief
   3. A belief the customer wants to buy DS
   4. A belief that the product is harmless.
   5. The pharmacy’s upselling policy

*

1. If no, why not? (it is possible to choose more than one reason)
   1. Insufficient knowledge about DS
   2. Do not believe DS to have positive effect
   3. Fear adverse events
   4. Fear interactions with PD

*

1. Do you give information on adverse events from DS including possible interactions?
   1. Yes
   2. No
   3. Sometimes

*

1. Independently of your pharmacy’s routines, do you ask customers about PD use when selling DS?
   1. Yes, always
   2. Only when I find there is a reason to do so because of the customer’s health
   3. Only for certain types of DS
   4. No

*If the answer is no, the respondents should go directly to Question 29.*

1. If you do ask, do you also check for potential interactions? If you do, which sources do you use for checking?

*

Open-ended question

*

1. Which option is best to ensure the correct and safe use of DS in persons with dementia? You can choose more than one alternative, prioritize your choices

*

- 1. Information from health authorities to the general population
  2. Changes in laws and regulations concerning DS
  3. Increased effort from GPs (ask all patients about use of DS and check for adverse events and interactions)
  4. Increased effort from home care services (convey information about the use of DS to GPs or pharmacists)
  5. Increased effort from pharmacies (check for interactions for all customers who buy DS, inform GPs)
  6. DS delivered in multidose drug-dispensing systems together with PD

1. What is your most important sources of information about DS? It is possible to choose more than one option.
   1. Textbooks, scientific publications
   2. From family and friends
   3. Web pages recommended by Norwegian health authorities and pharmaceutical research environments
   4. Courses on DS (post-school training/post-qualifying education)
   5. My professional education
   6. Product information/leaflets
   7. Media and magazines
2. If you have participated in courses on DS, who were responsible for this education. Were the goal of this education to increase upselling?

*

1. Does your pharmacy provide access to independent scientific information on the DS sold in your pharmacy?
   1. Yes, on all DS sold in the pharmacy
   2. On most products
   3. On a few products
   4. No
2. From which sources do you normally seek information about DS?

*

1. Do you agree with this statement: “Use of DS may have potentially harmful effects to the users’ health”?
   1. Yes
   2. No
   3. Do not know
2. Which of the following DS should not be taken together with Warfarin? You can choose more than one alternative
3. Top of Form
4. Bottom of Form
   1. Ginkgo biloba
   2. St John’s wort
   3. Salvia officinalis
   4. Echinacea
